# Bis(μ-2′-carboxyl­atobiphenyl-2-carboxylic acid-κ^2^ *O* ^2^:*O* ^2′^)bis­[(2,2′-bipyridine-κ^2^ *N*,*N*′)(2′-carboxyl­ato­biphenyl-2-carboxylic acid-κ*O* ^2′^)zinc(II)]

**DOI:** 10.1107/S1600536809045541

**Published:** 2009-11-04

**Authors:** Zhe An

**Affiliations:** a School of Chemistry and Life Science, Maoming University, Maoming 525000, People’s Republic of China

## Abstract

In the dimeric title compound, [Zn_2_(C_14_H_9_O_4_)_4_(C_10_H_8_N_2_)_2_], the Zn^II^ ions are penta­coordinated by one 2,2′-bipyridyl ligand and by three O atoms from three 2′-carboxyl­atobiphenyl-2-carboxylic acid ligands. Two of the 2′-carboxyl­atobiphenyl-2-carboxylic acid ligands act as bridging ligands and, together with two zinc(II) cations, produce an 18-membered ring system. The remaining 2′-carboxyl­atobiphenyl-2-carboxylic acid ligands work as monodentate ligands. The crystal packing diagram is consolidated by O—H⋯O hydrogen bonds.

## Related literature

For related structures of metal-organic frameworks incorporating zinc or lanthanides and dicarboxylic acids, see: Wan & Zhang (2003 ▶); Vodak *et al.* (2001 ▶).
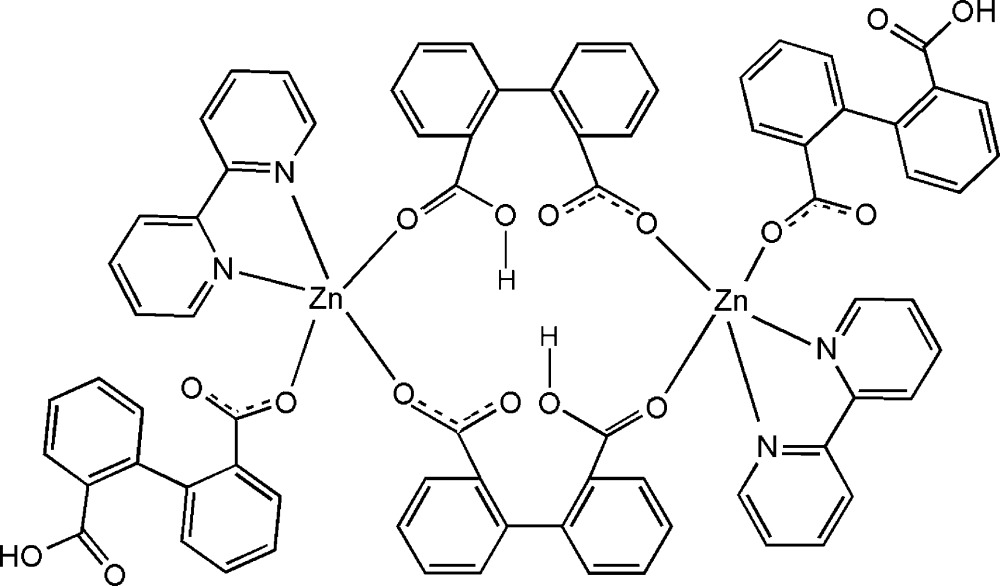


## Experimental

### 

#### Crystal data

[Zn_2_(C_14_H_9_O_4_)_4_(C_10_H_8_N_2_)_2_]
*M* *_r_* = 1407.96
Triclinic, 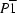

*a* = 10.8745 (4) Å
*b* = 11.6465 (4) Å
*c* = 14.0223 (5) Å
α = 103.094 (1)°
β = 112.773 (1)°
γ = 92.680 (1)°
*V* = 1576.9 (1) Å^3^
*Z* = 1
Mo *K*α radiation
μ = 0.84 mm^−1^
*T* = 293 K
0.12 × 0.10 × 0.08 mm

#### Data collection

Bruker SMART CCD area-detector diffractometer
Absorption correction: multi-scan (*SADABS*; Bruker, 2001 ▶) *T*_min_ = 0.906, *T*_max_ = 0.936
10807 measured reflections
5332 independent reflections
4820 reflections with *I* > 2σ(*I*)
*R*_int_ = 0.014

#### Refinement

*R*[*F* ^2^ > 2σ(*F* ^2^)] = 0.027
*wR*(*F* ^2^) = 0.098
*S* = 1.00
5332 reflections
444 parameters
H-atom parameters not refined
Δρ_max_ = 0.29 e Å^−3^
Δρ_min_ = −0.25 e Å^−3^

### 

Data collection: *SMART* (Bruker, 2005 ▶); cell refinement: *SAINT-Plus* (Bruker, 2005 ▶); data reduction: *SAINT-Plus*; program(s) used to solve structure: *SHELXS97* (Sheldrick, 2008 ▶); program(s) used to refine structure: *SHELXL97* (Sheldrick, 2008 ▶); molecular graphics: *XP* in *SHELXTL* (Sheldrick, 2008 ▶); software used to prepare material for publication: *SHELXL97*.

## Supplementary Material

Crystal structure: contains datablocks I, global. DOI: 10.1107/S1600536809045541/im2149sup1.cif

Structure factors: contains datablocks I. DOI: 10.1107/S1600536809045541/im2149Isup2.hkl

Additional supplementary materials:  crystallographic information; 3D view; checkCIF report

## Figures and Tables

**Table 1:** Hydrogen-bond geometry (Å, °)

*D*—H⋯*A*	*D*—H	H⋯*A*	*D*⋯*A*	*D*—H⋯*A*
O4—H4*A*⋯O2^i^	0.82	1.85	2.6545 (19)	167
O7—H7⋯O6	0.82	1.74	2.551 (2)	170

Symmetry code: (i) 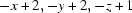.

**Fig. 1.**
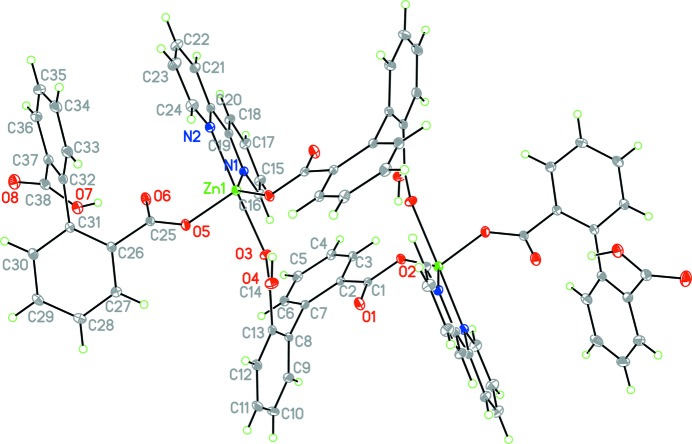
A view of the molecular structure of the title compound, showing the atomic numbering scheme and 50% probability displacement ellipsoids.

**Fig. 2.**
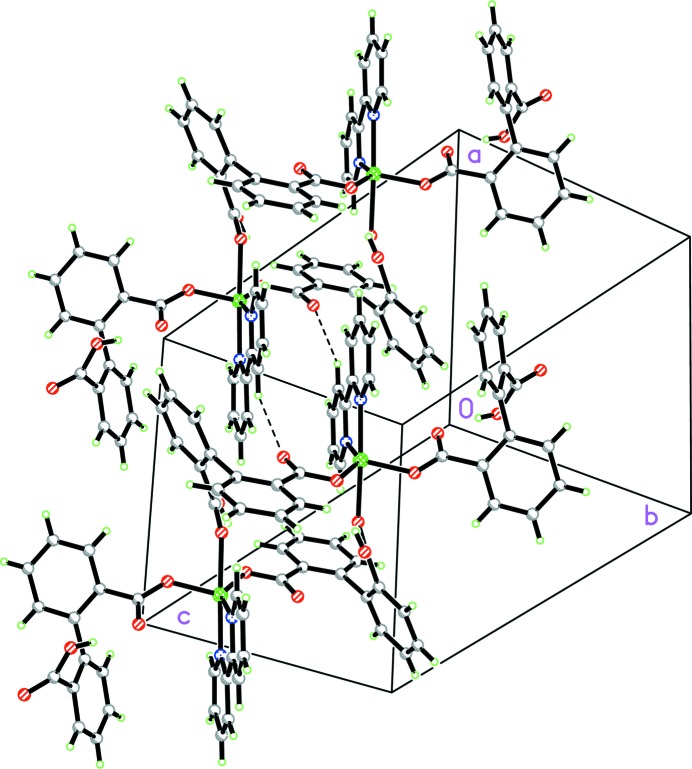
The packing diagram of the title compound.

## supplementary crystallographic information

### Comment

Aromatic dicarboxylic acids are widely used in the construction of coordination polymers due to their capability of acting as bridging and chelating ligands in various coordination modes (Wan & Zhang, 2003). The preparation of metal aromatic carboxylates under hydrothermal conditions has also been reported (Vodak *et al.*2001).

In the present paper, we describe the synthesis and structural characterization of the title compound.

As shown in figure 1, the Zn^II^ ions are pentacoordinated by one 2,2'-bipyridyl ligand and by three oxygen atoms from three 2'-carboxylatobiphenyl-2-carboxylic acid ligands. Two of the 2'-carboxylatobiphenyl-2-carboxylic acid ligands act as bridging ligands and together with two zinc(II) cations produce an 18 membered ring system. The Zn···Zn distance measures to 6.701 Å. The remaining 2'-carboxylatobiphenyl-2-carboxylic acid ligands work as monodentate ligands. The crystal packing diagram is consolidated by O—H···O hydrogen bonds. (Table 1, Figure 2).

### Experimental

A mixture of Zn(CH_3_COO)_2_ × 2 H_2_O (0.25 mmol), biphenyl-2,2'-dicarboxylic acid (0.2 mmol), 2,2'-bipyridyl (0.2 mmol), sodium hydroxide(0.5 mmol) and water (12 ml) was stirred for 30 min under air. The mixture was then transferred to a 25 ml Teflon-lined autoclave and was kept at 443 K for 72 h. Colorless prisms of the title compound were obtained from the reaction mixture after cooling to room temperature.

### Refinement

All H atoms were placed in calculated positions with C—H = 0.93Å for aromatic C-H functions and 0.82 Å for hydroxyl groups. Hydrogens were refined using a riding model with *U*_iso_(H) = 1.2*U*_eq_(C) and *U*_iso_(H) = 1.5*U*_eq_(O).

### Crystal data

**Table:**

[Zn_2_(C_14_H_9_O_4_)_4_(C_10_H_8_N_2_)_2_]	*Z* = 1
*M**_r_* = 1407.96	*F*(000) = 724
Triclinic, *P*1	*D*_x_ = 1.483 Mg m^−^^3^
Hall symbol: -P 1	Mo *K*α radiation, λ = 0.71073 Å
*a* = 10.8745 (4) Å	Cell parameters from 6287 reflections
*b* = 11.6465 (4) Å	θ = 2.5–28.2°
*c* = 14.0223 (5) Å	µ = 0.84 mm^−^^1^
α = 103.094 (1)°	*T* = 293 K
β = 112.773 (1)°	Block, translucent
γ = 92.680 (1)°	0.12 × 0.10 × 0.08 mm
*V* = 1576.9 (1) Å^3^	

### Data collection

**Table:**

Bruker SMART CCD area-detector diffractometer	5332 independent reflections
Radiation source: fine-focus sealed tube	4820 reflections with *I* > 2σ(*I*)
graphite	*R*_int_ = 0.014
φ and ω scans	θ_max_ = 25.0°, θ_min_ = 2.5°
Absorption correction: multi-scan (*SADABS*; Bruker, 2001)	*h* = −12→12
*T*_min_ = 0.906, *T*_max_ = 0.936	*k* = −13→13
10807 measured reflections	*l* = −16→16

### Refinement

**Table:**

Refinement on *F*^2^	Primary atom site location: structure-invariant direct methods
Least-squares matrix: full	Secondary atom site location: difference Fourier map
*R*[*F*^2^ > 2σ(*F*^2^)] = 0.027	Hydrogen site location: inferred from neighbouring sites
*wR*(*F*^2^) = 0.098	H-atom parameters not refined
*S* = 1.00	*w* = 1/[σ^2^(*F*_o_^2^) + (0.08*P*)^2^] where *P* = (*F*_o_^2^ + 2*F*_c_^2^)/3
5332 reflections	(Δ/σ)_max_ = 0.001
444 parameters	Δρ_max_ = 0.29 e Å^−^^3^
0 restraints	Δρ_min_ = −0.25 e Å^−^^3^

### Special details

**Table:**

Geometry. All esds (except the esd in the dihedral angle between two l.s. planes) are estimated using the full covariance matrix. The cell esds are taken into account individually in the estimation of esds in distances, angles and torsion angles; correlations between esds in cell parameters are only used when they are defined by crystal symmetry. An approximate (isotropic) treatment of cell esds is used for estimating esds involving l.s. planes.
Refinement. Refinement of F^2^ against ALL reflections. The weighted R-factor wR and goodness of fit S are based on F^2^, conventional R-factors R are based on F, with F set to zero for negative F^2^. The threshold expression of F^2^ > 2sigma(F^2^) is used only for calculating R-factors(gt) etc. and is not relevant to the choice of reflections for refinement. R-factors based on F^2^ are statistically about twice as large as those based on F, and R- factors based on ALL data will be even larger.

### Fractional atomic coordinates and isotropic or equivalent isotropic displacement parameters (Å^2^)

**Table:**

	*x*	*y*	*z*	*U*_iso_*/*U*_eq_	
Zn1	0.852166 (18)	0.917078 (17)	0.648679 (15)	0.03426 (10)	
C1	1.04604 (17)	0.86670 (15)	0.35850 (14)	0.0326 (4)	
C2	0.95130 (17)	0.75294 (15)	0.32443 (13)	0.0320 (4)	
C3	0.82737 (18)	0.73778 (17)	0.23642 (14)	0.0370 (4)	
H3	0.8065	0.7990	0.2029	0.044*	
C4	0.7356 (2)	0.63560 (18)	0.19791 (15)	0.0436 (5)	
H4	0.6547	0.6274	0.1386	0.052*	
C5	0.7648 (2)	0.54537 (18)	0.24809 (16)	0.0464 (5)	
H5	0.7027	0.4763	0.2237	0.056*	
C6	0.8865 (2)	0.55764 (17)	0.33480 (16)	0.0421 (4)	
H6	0.9054	0.4958	0.3677	0.050*	
C7	0.98164 (18)	0.65955 (15)	0.37432 (14)	0.0333 (4)	
C8	1.11438 (18)	0.65573 (16)	0.46245 (16)	0.0381 (4)	
C9	1.1866 (2)	0.56587 (19)	0.43731 (19)	0.0512 (5)	
H9	1.1517	0.5144	0.3686	0.061*	
C10	1.3082 (2)	0.5520 (2)	0.5122 (2)	0.0639 (7)	
H10	1.3538	0.4910	0.4940	0.077*	
C11	1.3621 (2)	0.6283 (2)	0.6136 (2)	0.0630 (7)	
H11	1.4452	0.6200	0.6636	0.076*	
C12	1.2928 (2)	0.7173 (2)	0.64123 (18)	0.0500 (5)	
H12	1.3292	0.7684	0.7101	0.060*	
C13	1.16833 (18)	0.73104 (17)	0.56643 (15)	0.0388 (4)	
C14	1.09428 (18)	0.82548 (17)	0.60017 (14)	0.0369 (4)	
C15	0.66830 (19)	0.74974 (17)	0.43258 (15)	0.0418 (4)	
H15	0.7471	0.7338	0.4242	0.050*	
C16	0.5473 (2)	0.68971 (19)	0.35408 (16)	0.0478 (5)	
H16	0.5443	0.6338	0.2939	0.057*	
C17	0.4306 (2)	0.71328 (19)	0.36542 (16)	0.0475 (5)	
H17	0.3474	0.6733	0.3131	0.057*	
C18	0.43798 (18)	0.79707 (18)	0.45546 (15)	0.0410 (4)	
H18	0.3598	0.8147	0.4641	0.049*	
C19	0.56275 (17)	0.85434 (15)	0.53254 (14)	0.0319 (4)	
C20	0.57917 (18)	0.94393 (15)	0.63252 (14)	0.0327 (4)	
C21	0.4710 (2)	0.97965 (18)	0.65380 (16)	0.0421 (4)	
H21	0.3831	0.9495	0.6042	0.050*	
C22	0.4958 (2)	1.0608 (2)	0.74987 (18)	0.0533 (5)	
H22	0.4245	1.0858	0.7661	0.064*	
C23	0.6265 (2)	1.1043 (2)	0.82138 (19)	0.0578 (6)	
H23	0.6450	1.1583	0.8870	0.069*	
C24	0.7292 (2)	1.06703 (19)	0.79452 (16)	0.0498 (5)	
H24	0.8178	1.0976	0.8425	0.060*	
C25	0.89081 (19)	0.77209 (16)	0.79160 (14)	0.0363 (4)	
C26	0.97304 (19)	0.72768 (16)	0.88630 (14)	0.0359 (4)	
C27	1.0903 (2)	0.68258 (19)	0.88947 (16)	0.0470 (5)	
H27	1.1207	0.6867	0.8366	0.056*	
C28	1.1621 (2)	0.63166 (19)	0.97031 (17)	0.0501 (5)	
H28	1.2391	0.5997	0.9707	0.060*	
C29	1.1194 (2)	0.62845 (18)	1.05020 (16)	0.0480 (5)	
H29	1.1679	0.5948	1.1050	0.058*	
C30	1.00411 (19)	0.67535 (17)	1.04919 (14)	0.0406 (4)	
H30	0.9764	0.6733	1.1039	0.049*	
C31	0.92915 (18)	0.72541 (15)	0.96747 (13)	0.0332 (4)	
C32	0.81105 (19)	0.78099 (16)	0.97435 (13)	0.0364 (4)	
C33	0.8325 (2)	0.90124 (19)	1.02957 (16)	0.0503 (5)	
H33	0.9165	0.9467	1.0534	0.060*	
C34	0.7298 (3)	0.9532 (2)	1.04906 (19)	0.0693 (8)	
H34	0.7447	1.0338	1.0844	0.083*	
C35	0.6045 (3)	0.8859 (3)	1.0162 (2)	0.0732 (8)	
H35	0.5368	0.9206	1.0316	0.088*	
C36	0.5812 (3)	0.7675 (2)	0.9608 (2)	0.0614 (6)	
H36	0.4974	0.7223	0.9383	0.074*	
C37	0.6829 (2)	0.71558 (19)	0.93839 (16)	0.0447 (5)	
C38	0.64762 (19)	0.58724 (19)	0.87503 (17)	0.0460 (5)	
N1	0.67766 (14)	0.83087 (13)	0.52131 (11)	0.0339 (3)	
N2	0.70656 (15)	0.98810 (13)	0.70158 (12)	0.0356 (3)	
O1	1.15618 (13)	0.88852 (13)	0.43496 (12)	0.0513 (4)	
O2	1.00560 (12)	0.94223 (11)	0.30197 (10)	0.0394 (3)	
O3	0.97207 (13)	0.81690 (13)	0.56208 (10)	0.0435 (3)	
O4	1.17363 (13)	0.91838 (13)	0.67693 (12)	0.0498 (4)	
H4A	1.1282	0.9697	0.6883	0.075*	
O5	0.95305 (13)	0.84733 (13)	0.76743 (11)	0.0459 (3)	
O6	0.76884 (14)	0.73196 (13)	0.74187 (11)	0.0496 (4)	
O7	0.68307 (16)	0.55831 (13)	0.79382 (12)	0.0510 (4)	
H7	0.7080	0.6191	0.7820	0.076*	
O8	0.58752 (17)	0.51275 (17)	0.89517 (14)	0.0684 (5)	

### Atomic displacement parameters (Å^2^)

**Table:**

	*U*^11^	*U*^22^	*U*^33^	*U*^12^	*U*^13^	*U*^23^
Zn1	0.02564 (15)	0.03895 (15)	0.03585 (15)	−0.00024 (10)	0.00903 (10)	0.01293 (10)
C1	0.0297 (9)	0.0355 (9)	0.0371 (9)	0.0049 (7)	0.0174 (8)	0.0115 (7)
C2	0.0310 (9)	0.0354 (9)	0.0337 (9)	0.0048 (7)	0.0176 (7)	0.0093 (7)
C3	0.0361 (10)	0.0421 (10)	0.0343 (9)	0.0021 (8)	0.0152 (8)	0.0120 (8)
C4	0.0379 (11)	0.0529 (12)	0.0340 (10)	−0.0039 (9)	0.0124 (8)	0.0066 (8)
C5	0.0459 (12)	0.0391 (10)	0.0493 (11)	−0.0092 (9)	0.0220 (10)	0.0012 (9)
C6	0.0480 (11)	0.0333 (10)	0.0487 (11)	0.0038 (9)	0.0234 (9)	0.0118 (8)
C7	0.0355 (9)	0.0315 (9)	0.0379 (9)	0.0046 (7)	0.0201 (8)	0.0094 (7)
C8	0.0354 (10)	0.0377 (10)	0.0510 (11)	0.0093 (8)	0.0209 (9)	0.0235 (8)
C9	0.0534 (13)	0.0434 (11)	0.0707 (14)	0.0173 (10)	0.0340 (11)	0.0238 (10)
C10	0.0569 (14)	0.0620 (14)	0.097 (2)	0.0334 (13)	0.0414 (14)	0.0428 (15)
C11	0.0408 (12)	0.0788 (17)	0.0857 (18)	0.0242 (12)	0.0231 (12)	0.0542 (15)
C12	0.0348 (11)	0.0653 (14)	0.0557 (12)	0.0089 (10)	0.0144 (9)	0.0343 (11)
C13	0.0305 (10)	0.0461 (11)	0.0480 (11)	0.0069 (8)	0.0166 (8)	0.0268 (9)
C14	0.0309 (10)	0.0471 (11)	0.0340 (9)	0.0030 (8)	0.0121 (8)	0.0159 (8)
C15	0.0359 (10)	0.0464 (11)	0.0423 (10)	0.0064 (8)	0.0185 (8)	0.0058 (8)
C16	0.0420 (11)	0.0499 (12)	0.0430 (11)	−0.0009 (9)	0.0164 (9)	−0.0003 (9)
C17	0.0334 (10)	0.0535 (12)	0.0421 (11)	−0.0071 (9)	0.0087 (8)	0.0024 (9)
C18	0.0276 (9)	0.0482 (11)	0.0453 (11)	0.0006 (8)	0.0154 (8)	0.0087 (9)
C19	0.0283 (9)	0.0342 (9)	0.0351 (9)	0.0016 (7)	0.0132 (7)	0.0129 (7)
C20	0.0317 (9)	0.0318 (9)	0.0381 (9)	0.0006 (7)	0.0158 (8)	0.0140 (7)
C21	0.0346 (10)	0.0453 (11)	0.0465 (11)	0.0007 (8)	0.0191 (8)	0.0091 (9)
C22	0.0507 (13)	0.0569 (13)	0.0563 (13)	0.0041 (10)	0.0320 (11)	0.0049 (10)
C23	0.0617 (14)	0.0565 (13)	0.0489 (12)	0.0003 (11)	0.0277 (11)	−0.0053 (10)
C24	0.0448 (12)	0.0495 (12)	0.0429 (11)	−0.0019 (10)	0.0119 (9)	0.0019 (9)
C25	0.0442 (11)	0.0356 (9)	0.0334 (9)	0.0102 (8)	0.0186 (8)	0.0115 (7)
C26	0.0391 (10)	0.0312 (9)	0.0361 (9)	0.0043 (8)	0.0133 (8)	0.0105 (7)
C27	0.0465 (12)	0.0519 (12)	0.0475 (11)	0.0127 (10)	0.0222 (10)	0.0163 (9)
C28	0.0425 (12)	0.0478 (12)	0.0552 (13)	0.0157 (10)	0.0137 (10)	0.0143 (10)
C29	0.0479 (12)	0.0449 (11)	0.0414 (11)	0.0107 (10)	0.0053 (9)	0.0158 (9)
C30	0.0441 (11)	0.0400 (10)	0.0317 (9)	0.0043 (9)	0.0092 (8)	0.0100 (8)
C31	0.0348 (10)	0.0286 (8)	0.0290 (8)	−0.0001 (7)	0.0073 (7)	0.0053 (7)
C32	0.0430 (11)	0.0382 (10)	0.0270 (9)	0.0089 (8)	0.0113 (8)	0.0116 (7)
C33	0.0672 (14)	0.0443 (11)	0.0362 (10)	0.0150 (10)	0.0174 (10)	0.0101 (8)
C34	0.109 (2)	0.0605 (15)	0.0535 (14)	0.0457 (16)	0.0412 (15)	0.0235 (12)
C35	0.085 (2)	0.092 (2)	0.0759 (17)	0.0533 (18)	0.0524 (16)	0.0417 (16)
C36	0.0525 (14)	0.0817 (17)	0.0691 (15)	0.0294 (13)	0.0319 (12)	0.0390 (13)
C37	0.0417 (11)	0.0555 (12)	0.0406 (10)	0.0136 (10)	0.0142 (9)	0.0230 (9)
C38	0.0310 (10)	0.0513 (12)	0.0474 (11)	0.0012 (9)	0.0042 (8)	0.0201 (9)
N1	0.0287 (8)	0.0384 (8)	0.0361 (8)	0.0033 (6)	0.0136 (6)	0.0124 (6)
N2	0.0317 (8)	0.0346 (8)	0.0380 (8)	−0.0005 (6)	0.0123 (6)	0.0094 (6)
O1	0.0352 (8)	0.0526 (8)	0.0558 (9)	−0.0070 (6)	0.0030 (7)	0.0260 (7)
O2	0.0342 (7)	0.0366 (7)	0.0483 (8)	0.0024 (5)	0.0130 (6)	0.0203 (6)
O3	0.0279 (7)	0.0572 (8)	0.0404 (7)	0.0044 (6)	0.0132 (6)	0.0052 (6)
O4	0.0307 (7)	0.0553 (9)	0.0516 (8)	0.0009 (6)	0.0108 (6)	0.0038 (7)
O5	0.0429 (8)	0.0544 (8)	0.0501 (8)	0.0091 (7)	0.0206 (6)	0.0289 (7)
O6	0.0453 (9)	0.0557 (9)	0.0412 (7)	−0.0014 (7)	0.0069 (6)	0.0218 (7)
O7	0.0520 (9)	0.0432 (8)	0.0482 (8)	−0.0056 (7)	0.0126 (7)	0.0108 (6)
O8	0.0558 (10)	0.0727 (11)	0.0721 (11)	−0.0142 (9)	0.0195 (8)	0.0273 (9)

### Geometric parameters (Å, °)

**Table:**

Zn1—O5	1.9795 (12)	C19—C20	1.487 (2)
Zn1—O2^i^	1.9941 (13)	C20—N2	1.338 (2)
Zn1—N1	2.0456 (15)	C20—C21	1.381 (3)
Zn1—N2	2.1203 (15)	C21—C22	1.378 (3)
Zn1—O3	2.2940 (13)	C21—H21	0.9300
C1—O1	1.226 (2)	C22—C23	1.372 (3)
C1—O2	1.295 (2)	C22—H22	0.9300
C1—C2	1.500 (3)	C23—C24	1.369 (3)
C2—C7	1.407 (2)	C23—H23	0.9300
C2—C3	1.401 (2)	C24—N2	1.339 (3)
C3—C4	1.373 (3)	C24—H24	0.9300
C3—H3	0.9300	C25—O6	1.242 (2)
C4—C5	1.376 (3)	C25—O5	1.264 (2)
C4—H4	0.9300	C25—C26	1.505 (2)
C5—C6	1.382 (3)	C26—C27	1.390 (3)
C5—H5	0.9300	C26—C31	1.399 (3)
C6—C7	1.389 (3)	C27—C28	1.382 (3)
C6—H6	0.9300	C27—H27	0.9300
C7—C8	1.504 (2)	C28—C29	1.376 (3)
C8—C13	1.396 (3)	C28—H28	0.9300
C8—C9	1.400 (3)	C29—C30	1.387 (3)
C9—C10	1.378 (3)	C29—H29	0.9300
C9—H9	0.9300	C30—C31	1.393 (2)
C10—C11	1.374 (4)	C30—H30	0.9300
C10—H10	0.9300	C31—C32	1.491 (3)
C11—C12	1.382 (3)	C32—C37	1.400 (3)
C11—H11	0.9300	C32—C33	1.398 (3)
C12—C13	1.398 (3)	C33—C34	1.383 (3)
C12—H12	0.9300	C33—H33	0.9300
C13—C14	1.491 (3)	C34—C35	1.391 (4)
C14—O3	1.215 (2)	C34—H34	0.9300
C14—O4	1.319 (2)	C35—C36	1.378 (4)
C15—N1	1.346 (2)	C35—H35	0.9300
C15—C16	1.370 (3)	C36—C37	1.390 (3)
C15—H15	0.9300	C36—H36	0.9300
C16—C17	1.371 (3)	C37—C38	1.500 (3)
C16—H16	0.9300	C38—O8	1.208 (3)
C17—C18	1.382 (3)	C38—O7	1.318 (3)
C17—H17	0.9300	O2—Zn1^i^	1.9941 (13)
C18—C19	1.381 (3)	O4—H4A	0.8200
C18—H18	0.9300	O7—H7	0.8200
C19—N1	1.351 (2)		
			
O5—Zn1—O2^i^	94.20 (5)	N2—C20—C19	115.57 (15)
O5—Zn1—N1	123.40 (6)	C21—C20—C19	122.76 (16)
O2^i^—Zn1—N1	141.68 (6)	C22—C21—C20	118.79 (19)
O5—Zn1—N2	100.24 (6)	C22—C21—H21	120.6
O2^i^—Zn1—N2	102.90 (6)	C20—C21—H21	120.6
N1—Zn1—N2	79.34 (6)	C23—C22—C21	119.44 (19)
O5—Zn1—O3	85.49 (5)	C23—C22—H22	120.3
O2^i^—Zn1—O3	86.73 (5)	C21—C22—H22	120.3
N1—Zn1—O3	88.97 (5)	C24—C23—C22	118.9 (2)
N2—Zn1—O3	168.28 (5)	C24—C23—H23	120.6
O1—C1—O2	120.88 (17)	C22—C23—H23	120.6
O1—C1—C2	122.88 (16)	N2—C24—C23	122.3 (2)
O2—C1—C2	116.24 (15)	N2—C24—H24	118.9
C7—C2—C3	118.33 (17)	C23—C24—H24	118.9
C7—C2—C1	123.57 (16)	O6—C25—O5	124.57 (17)
C3—C2—C1	118.08 (15)	O6—C25—C26	118.81 (17)
C4—C3—C2	122.22 (18)	O5—C25—C26	116.61 (17)
C4—C3—H3	118.9	C27—C26—C31	119.98 (16)
C2—C3—H3	118.9	C27—C26—C25	119.06 (16)
C3—C4—C5	119.23 (18)	C31—C26—C25	120.87 (16)
C3—C4—H4	120.4	C26—C27—C28	120.69 (19)
C5—C4—H4	120.4	C26—C27—H27	119.7
C6—C5—C4	119.80 (19)	C28—C27—H27	119.7
C6—C5—H5	120.1	C29—C28—C27	119.74 (18)
C4—C5—H5	120.1	C29—C28—H28	120.1
C5—C6—C7	121.99 (18)	C27—C28—H28	120.1
C5—C6—H6	119.0	C28—C29—C30	120.14 (18)
C7—C6—H6	119.0	C28—C29—H29	119.9
C6—C7—C2	118.41 (17)	C30—C29—H29	119.9
C6—C7—C8	116.00 (16)	C31—C30—C29	120.97 (18)
C2—C7—C8	125.39 (16)	C31—C30—H30	119.5
C13—C8—C9	118.14 (18)	C29—C30—H30	119.5
C13—C8—C7	125.70 (16)	C30—C31—C26	118.45 (17)
C9—C8—C7	116.13 (18)	C30—C31—C32	118.44 (16)
C10—C9—C8	121.4 (2)	C26—C31—C32	123.00 (15)
C10—C9—H9	119.3	C37—C32—C33	118.25 (19)
C8—C9—H9	119.3	C37—C32—C31	122.99 (16)
C11—C10—C9	120.1 (2)	C33—C32—C31	118.39 (18)
C11—C10—H10	119.9	C34—C33—C32	120.6 (2)
C9—C10—H10	119.9	C34—C33—H33	119.7
C10—C11—C12	119.9 (2)	C32—C33—H33	119.7
C10—C11—H11	120.0	C33—C34—C35	120.5 (2)
C12—C11—H11	120.0	C33—C34—H34	119.8
C13—C12—C11	120.5 (2)	C35—C34—H34	119.8
C13—C12—H12	119.8	C34—C35—C36	119.8 (2)
C11—C12—H12	119.8	C34—C35—H35	120.1
C8—C13—C12	119.93 (18)	C36—C35—H35	120.1
C8—C13—C14	121.07 (16)	C37—C36—C35	120.0 (3)
C12—C13—C14	118.99 (18)	C37—C36—H36	120.0
O3—C14—O4	122.94 (17)	C35—C36—H36	120.0
O3—C14—C13	123.24 (18)	C32—C37—C36	120.9 (2)
O4—C14—C13	113.81 (15)	C32—C37—C38	122.62 (17)
N1—C15—C16	122.57 (17)	C36—C37—C38	116.5 (2)
N1—C15—H15	118.7	O8—C38—O7	120.5 (2)
C16—C15—H15	118.7	O8—C38—C37	122.5 (2)
C15—C16—C17	119.07 (18)	O7—C38—C37	117.02 (18)
C15—C16—H16	120.5	C15—N1—C19	118.43 (16)
C17—C16—H16	120.5	C15—N1—Zn1	126.19 (12)
C16—C17—C18	119.23 (18)	C19—N1—Zn1	115.26 (12)
C16—C17—H17	120.4	C20—N2—C24	118.92 (16)
C18—C17—H17	120.4	C20—N2—Zn1	113.63 (11)
C19—C18—C17	119.30 (17)	C24—N2—Zn1	127.42 (14)
C19—C18—H18	120.4	C1—O2—Zn1^i^	111.14 (11)
C17—C18—H18	120.3	C14—O3—Zn1	123.99 (12)
N1—C19—C18	121.39 (16)	C14—O4—H4A	109.5
N1—C19—C20	116.12 (15)	C25—O5—Zn1	119.66 (12)
C18—C19—C20	122.49 (16)	C38—O7—H7	109.5
N2—C20—C21	121.67 (17)		

Symmetry codes: (i) −*x*+2, −*y*+2, −*z*+1.

### Hydrogen-bond geometry (Å, °)

**Table:**

*D*—H···*A*	*D*—H	H···*A*	*D*···*A*	*D*—H···*A*
O4—H4A···O2^i^	0.82	1.85	2.6545 (19)	167
O7—H7···O6	0.82	1.74	2.551 (2)	170

Symmetry codes: (i) −*x*+2, −*y*+2, −*z*+1.
